# Effect of Stay-at-Home orders and other COVID-related policies on trauma hospitalization rates and disparities in the USA: a statewide time-series analysis

**DOI:** 10.1186/s40621-022-00409-2

**Published:** 2022-11-21

**Authors:** Paula D. Strassle, Alan C. Kinlaw, Jamie S. Ko, Stephanie M. Quintero, Jackie Bonilla, Madison Ponder, Anna María Nápoles, Sharon E. Schiro

**Affiliations:** 1 Division of Intramural Research, National Institute on Minority Health and Health Disparities, National Institutes of Health, Bethesda, MD USA; 2 Division of Pharmaceutical Outcomes and Policy, University of North Carolina School of Pharmacy, Chapel Hill, NC USA; 3 Cecil G. Sheps Center for Health Services Research, University of North Carolina at Chapel Hill, Chapel Hill, NC USA; 4 Department of Epidemiology, Rollins School of Public Health, Emory University, Atlanta, GA USA; 5 Department of Epidemiology, Gillings School of Public Health, University of North Carolina at Chapel Hill, Chapel Hill, NC USA; 6 Division of General, Acute Care, and Trauma Surgery, Department of Surgery, University of North Carolina at Chapel Hill, Chapel Hill, NC USA

**Keywords:** Stay-at-Home, Trauma disparities, Assault, Motor vehicle collisions

## Abstract

**Background:**

To combat the coronavirus pandemic, states implemented several public health policies to reduce infection and transmission. Increasing evidence suggests that these prevention strategies also have had a profound impact on non-COVID healthcare utilization. The goal of this study was to determine the impact of a statewide Stay-at-Home order and other COVID-related policies on trauma hospitalizations, stratified by race/ethnicity, age, and sex.

**Methods:**

We used the North Carolina Trauma Registry, a statewide registry of trauma hospitalizations for 18 hospitals across North Carolina, including all North Carolina trauma centers, to calculate weekly rates of assault, self-inflicted, unintentional motor vehicle collision (MVC), and other unintentional injury hospitalizations between January 1, 2019, and December 31, 2020. Interrupted time-series design and segmented linear regression were used to estimate changes in hospitalization rates after several COVID-related executive orders, overall and stratified by race/ethnicity, age, and sex. Changes in hospitalization rates were assessed after 1) USA declaration of a public health emergency; 2) North Carolina statewide Stay-at-Home order; 3) Stay-at-Home order lifted with restrictions (Phase 2: Safer-at-Home); and 4) further lifting of restrictions (Phase 2.5: Safer-at-Home).

**Results:**

There were 70,478 trauma hospitalizations in North Carolina, 2019–2020. In 2020, median age was 53 years old and 59% were male. Assault hospitalization rates (per 1,000,000 NC residents) increased after the Stay-at-Home order, but substantial increases were only observed among Black/African American residents (weekly trend change = 1.147, 95% CI = 0.634 to 1.662) and 18–44-year-old males (weekly trend change = 1.708, 95% CI = 0.870 to 2.545). After major restrictions were lifted, assault rates decreased but remained elevated compared to pre-COVID levels. Unintentional non-MVC injury hospitalizations decreased after the USA declared a public health emergency, especially among women ≥ 65 years old (weekly trend change = -4.010, 95% CI = -6.166 to -1.855), but returned to pre-pandemic levels within several months.

**Conclusions:**

Statewide Stay-at-Home orders placed Black/African American residents at higher risk of assault hospitalizations, exacerbating pre-existing disparities. Males 18–44 years old were also at higher risk of assault hospitalization. Fear of COVID-19 may have led to decreases in unintentional non-MVC hospitalization rates, particularly among older females. Policy makers must anticipate policy-related harms that may disproportionately affect already disadvantaged communities and develop mitigation approaches.

**Supplementary Information:**

The online version contains supplementary material available at 10.1186/s40621-022-00409-2.

## Introduction

To combat the coronavirus pandemic, many countries implemented lockdowns and Stay-at-Home orders in 2020 to reduce transmission (Woskie et al. 2021); in the USA, these policies were implemented on a state-by-state basis (Wellenius et al. 2021). While these orders had a relatively positive impact on reducing COVID-19 infections (Woskie et al. 2021; Wellenius et al. 2021; Fraser et al. 2021), increasing evidence suggests that these prevention strategies also have had a profound impact on non-COVID healthcare utilization. For instance, Stay-at-Home orders in the USA have been associated with decreases in emergency department (ED) visits (Jeffery et al. 2020; Keyes et al. 2021), trauma admissions (Chiba et al. 2021; Kamine et al. 2020), and motor vehicle collisions (MVCs) (Chiba et al. 2021; Devarakonda et al. 2021; Sherman et al. 2021; Sutherland et al. 2020) as well as increases in suicide/suicidal attempts (Chiba et al. 2021; Czeisler et al. 2020; Hay et al. 2021; Hill et al. 2021), firearm injuries (Chiba et al. 2021; Sherman et al. 2021; Hatchimonji et al. 2020; Yeates et al. 2021), and domestic violence/child abuse (Kovler et al. 2021; Evans et al. 2020; Hsu and Henke 2021).

Despite trauma patients having presumed equal access to healthcare and the highly protocolized nature of trauma management plans, racial and ethnic minorities in the USA consistently had worse outcomes after traumatic injury prior to the COVID-19 pandemic (Chun Fat et al. 2019; Jager et al. 2019). Minority and low income individuals in the USA were also more likely to have public-facing occupations that required them to continue to work in person during the pandemic and Stay-at-Home orders (Yancy 2020; Webb Hooper et al. 2020). Minority and low-income individuals are also more likely to have crowded living conditions (Webb Hooper et al. 2020), which may place them at higher risk of domestic violence and other trauma during the pandemic. For these reasons and others, it is possible that Stay-at-Home orders and other COVID-related policies exacerbated known racial/ethnic disparities in trauma and non-COVID-related hospitalizations. Gaining a better understanding of the burden and potential exacerbation of traumatic injury disparities in the USA during the pandemic is necessary to inform and appropriately address mitigation efforts and related policies.

Thus, the goal of this study was to assess changes in traumatic injury hospitalization rates during the first year of the pandemic in the USA and assess how Stay-at-Home orders or COVID-related policies were associated with these changes. We were also interested in assessing whether the potential impact of these policies was similar across race/ethnicity, age, and sex.

## Methods

We used data from the North Carolina Trauma Registry (NCTR), a statewide registry and cooperative effort between 18 North Carolina hospitals, including all 17 North Carolina trauma centers (6 Level I, 3 Level II, and 8 Level III hospitals) and the North Carolina Office of Emergency Medical Services (NCOEMS) (Office of Emergency Medical Services, North Carolina Division of Health Service Regulation; Thomason 2008). This registry, which has been in place since 1987, collects near real-time information using standardized data definitions based on the National Trauma Registry of the American College of Surgeons and designated NCTR chart abstractors (Office of Emergency Medical Services, North Carolina Division of Health Service Regulation; Thomason 2008). The NCTR includes all hospitalizations where a patient is diagnosed with a traumatic injury (ICD-10-CM: S00-S99, T07, T14, T20-T28, T30-T32, T71, T79.A1-T79.A9) and is admitted to the hospital, taken to the operating room from the emergency department, transferred, or dies due to their injury. Unplanned readmissions within 30 days of the initial injury are also included.

We included all trauma hospitalizations that occurred between January 1, 2019, and December 31, 2020. To account for variation between weekday and weekend hospitalization rates, we calculated the weekly hospitalization rates for traumatic injuries per 1,000,000 North Carolina residents between January 6, 2019, and December 26, 2020. Admissions that occurred during partial weeks (January 1–5, 2019 and December 27–31, 2020) were excluded from modeling to avoid introducing bias due to underestimation (486 and 393 hospitalizations, respectively; in 2019, there were an average of 682 [SD 61.9] trauma hospitalizations per week). North Carolina population counts for 2019 were obtained from the North Carolina Office of State Budget and Management (North Carolina Office of State Budget and Management) and were used for both 2019 and 2020 hospitalization rate calculations.

Trauma admissions were classified by injury intent and mechanism into four categories—assault, self-inflicted, unintentional MVC (including MVC-bicyclist and MVC-pedestrian injuries), and unintentional non-MVC—using the ICD-10-CM code framework from the National Center for Health Statistics and National Center for Injury Prevention and Control (Hedegaard et al. 2019).

Data on race and ethnicity were used to categorize hospitalized patients as non-Hispanic Black/African American, Hispanic/Latino, non-Hispanic White, and non-Hispanic other race. Hispanic/Latino patients were classified as Hispanic/Latino, regardless of what was reported as their race. Other race included American Indian (n = 513 hospitalizations), Asian (n = 596 hospitalizations), Pacific Islander (n = 87 hospitalizations), multiracial (n = 202 hospitalizations), and those who listed “other” race (n = 1,048 hospitalizations). Race and ethnicity were self-reported by the patient (or family member) if they were present and capable; otherwise, it was based on staff designation in the electronic medical record.

COVID-related policies of interest included: USA declaration of a public health emergency (1/31/2020), the North Carolina statewide Stay-at-Home order (3/30/2020), an initial lifting of the Stay-at-Home order with restrictions (Phase 2: Safer-at-Home, 5/22/2020), and the further lifting of Stay-at-Home restrictions (Phase 2.5: Safer-at-Home, 9/4/2020), Additional file 1: Table S1. Policies were assigned to the week of their effective date. Other statewide executive orders that were not included in analyses were North Carolina declaring a state of emergency, statewide closure of K-12 public schools, Phases 1 and 3 of lifting the statewide Stay-at-Home orders, and the modified Stay-at-Home order issued before the 2020 holidays. These orders were not included in analyses because either the order made relatively small changes to existing orders (e.g., Phase 1 lifting of Stay-at-Home orders) or it occurred within several weeks of a prior order that we believed would be more salient (e.g., North Carolina declaring a state of emergency).

Differences in patient demographics and clinical characteristics among patients admitted for traumatic injuries between 2019 and 2020 were compared using standardized differences. An absolute difference > 0.20 was considered meaningful.

We conducted a natural experiment using an interrupted time-series design and segmented linear regression (Wagner et al. 2002; Kontopantelis et al. 2015). Using ordinary least squares, we conducted injury intent and mechanism-specific segmented linear regression models to estimate the trend in trauma hospitalization rates between each pair of interruptions. To reduce error in our model, we used a transformed cosine periodic function to control for potential seasonal fluctuations in hospitalization rates (Brookhart and Rothman 2008). To account for autocorrelation over time, we used Durbin–Watson tests (α = 0.05) to specify autoregressive parameters in our models for lags up to 60 weeks. We hypothesized that COVID-related policies would gradually impact trauma hospitalizations rates, and that no level changes (i.e., intercept changes) would be observed. To test this hypothesis, we included both level and slope changes in our initial overall models; however, all intercept changes were found to be not statistically significant (p > 0.05 for all). Therefore, based on our a priori-hypothesis and these findings, we decided to exclude intercept change parameters in our models and only model gradual changes in traumatic injury hospitalization rates. Model parameters for all analyses are reported in Additional file 1: Tables S2–S5.

Because no significant trend changes in hospitalization rates were seen prior to the pandemic, the average weekly rate of hospitalizations for this time period was estimated by taking the mean of all estimated weekly rates prior to the USA declaration of a public health emergency. The same methods were used to estimate rates during other time intervals if no significant trend changes were seen. Post-policy slopes were calculated by summing the slope after the policy of interest (e.g., Stay-at-Home order) with the preceding slopes (e.g., pre-pandemic slope and slope after the USA declared a public health emergency). (Wagner et al. 2002) 95% confidence intervals were calculated using the approximate standard errors generated for each post-policy slope.

The same methods were used to estimate race/ethnicity-, age-, and sex-specific hospitalization rates for assaults, MVCs, and other unintentional injuries. Due to low overall rates, race/ethnicity and age/sex-stratified models for self-inflicted injuries were not performed.

All analyses were performed using SAS version 9.4 (SAS Inc., Cary, North Carolina). All rates (and changes in rates) are reported as weekly rates (or weekly changes in rates) per 1,000,000 North Carolina residents. This study was deemed exempt by the University of North Carolina (IRB# 20–2117) and National Institutes of Health (IRB# 000,330) Institutional Review Boards.

## Results

Between 2019 and 2020, there were 70,478 trauma hospitalizations at participating sites (including all trauma centers); 43.6% (n = 30,712) occurred after COVID-19 was declared a USA public health emergency. In 2020, there were 354 confirmed and 5,543 suspected COVID-19 cases (16.9%) among hospitalized trauma patients. Demographics and clinical characteristics remained relatively consistent between 2019 and 2020, Table 1. The most common types of trauma admissions by intent/mechanism and year were unintentional non-MVC (2019: 60.5%; 2020: 59.6%), followed by unintentional MVCs (2019: 29.0%; 2020: 28.8%), assaults (2019: 9.3%; 2020: 10.4%), and then self-inflicted injuries (1.2% in both years). The majority of unintentional non-MVC hospitalizations between 2019 and 2020 were falls (n = 32,092, 77.4%).

**Table 1:** Demographics and clinical characteristics of trauma hospitalizations captured in the North Carolina Trauma Registry between 2019 and 2020, stratified by year

	2019		2020		SD^a^
	N	(%)	N	(%)	
Total, N	35,616		34,862		–
Age, years, med (IQR)	54	(28, 74)	53	(29, 74)	0.01
Age group, n (%)					
0–17	3,898	(10.9)	3,468	(9.9)	0.03
18–44	10,659	(29.9)	11,061	(31.7)	0.04
45–64	7,618	(21.4)	7,413	(21.3)	0.00
≥ 65	13,441	(37.7)	12,920	(37.1)	0.01
Male, n (%)	20,474	(57.5)	20,569	(59.0)	0.03
Race/ethnicity, n (%)					
American Indian	234	(0.7)	279	(0.8)	0.02
Asian	332	(0.9)	264	(0.8)	0.02
Black/African American	7,516	(21.3)	7,888	(22.9)	0.04
Hispanic/Latino	2,005	(5.7)	2,062	(6.0)	0.01
White	24,497	(69.6)	23,352	(67.7)	0.04
Other^b^	526	(1.5)	522	(1.5)	0.00
Multiracial	94	(0.3)	108	(0.3)	0.00
Missing	412		387		–
Primary payer, n (%)					
Any private insurance	10,862	(30.5)	9,954	(28.6)	0.04
Medicare/Medicaid only	16,010	(45.0)	15,785	(45.4)	0.01
Self-pay	5,629	(15.8)	5,981	(17.2)	0.04
Other^c^	3,061	(8.6)	3,066	(8.8)	0.01
Transferred to center, n (%)	10,578	(31.2)	9,323	(29.7)	0.03
ISS, med (IQR)	9	(4, 11)	9	(4, 13)	0.03
Mechanism, n (%)					
Assault	3,253	(9.3)	3,565	(10.4)	0.04
Self-inflicted	434	(1.2)	413	(1.2)	0.00
Unintentional	31,223	(89.4)	30,168	(88.4)	0.03
MVC^d^	10,116	(29.0)	9,817	(28.8)	0.01
Non-MVC	21,107	(60.5)	20,351	(59.6)	0.02
Undetermined	706		716		–
ED LOS, hours, med (IQR)	4.5	(2.8, 6.8)	4.6	(2.8, 7.2)	0.09
LOS, days, med (IQR)	3	(1, 6)	3	(1, 6)	0.03
ICU LOS^e^, days, med (IQR)	2	(1, 4)	2	(0, 4)	0.01
Discharge disposition, n (%)					
Routine/home	22,902	(65.6)	23,324	(68.7)	0.06
Long-term care^f^	8,865	(25.4)	7,488	(22.0)	0.00
Transferred^g^	1,878	(5.4)	1,815	(5.3)	0.08
Died	1,260	(3.6)	1,345	(4.0)	0.02
Missing^h^	711		890		–
COVID-19 infection, n (%)					
Confirmed	N/A		354	(1.0)	–
Suspected	N/A		5,543	(15.9)	–

SD, standardized difference; med, median; IQR, interquartile range; ISS, injury severity score; MVC, motor-vehicle collisions; ED, emergency department; LOS, length of stay; ICU, intensive care unit

^a^Absolute standardized difference (SD) comparing demographics and clinical characteristics between 2019 and 2020; an SD > 0.20 was considered meaningfully different

^b^Other race includes Other race and Hawaiian/Pacific Islander; race was collapsed due to small cell sizes

^c^Other insurance types include worker’s compensation, other government insurance, Champus, and not billed

^d^Include all MVC-related (e.g., MVC-bicyclist, MVC-pedestrian), motorcyclist, and other transport accidents

^e^Among those admitted to ICU (n = 20,827)

^f^Long-term care includes: hospice, long-term care facility, nursing home, rehabilitation facility, skilled nursing facility (SNF)

^g^Transfers to: acute care facilities, burn center, mental health facility, other trauma center, and transferred (unspecified)

^h^Includes individuals who left against medical advice (n = 559)

Prior to the COVID-19 pandemic, the weekly hospitalization rates of assaults (average rate = 5.93, weekly trend change = 0.001 [95% CI = -0.005 to 0.007], p = 0.73), self-inflicted injuries (average rate = 0.80, weekly trend change = 0.002 [95% CI = -0.001 to 0.005], p = 0.27), unintentional MVCs (average rate = 18.39, weekly trend change = 0.015 [95% CI = -0.037 to 0.067], p = 0.57), and unintentional non-MVCs (average rate = 38.54, weekly trend change = 0.012 [95% CI = -0.033 to 0.056], p = 0.61) were stable.

**Fig. 1:**
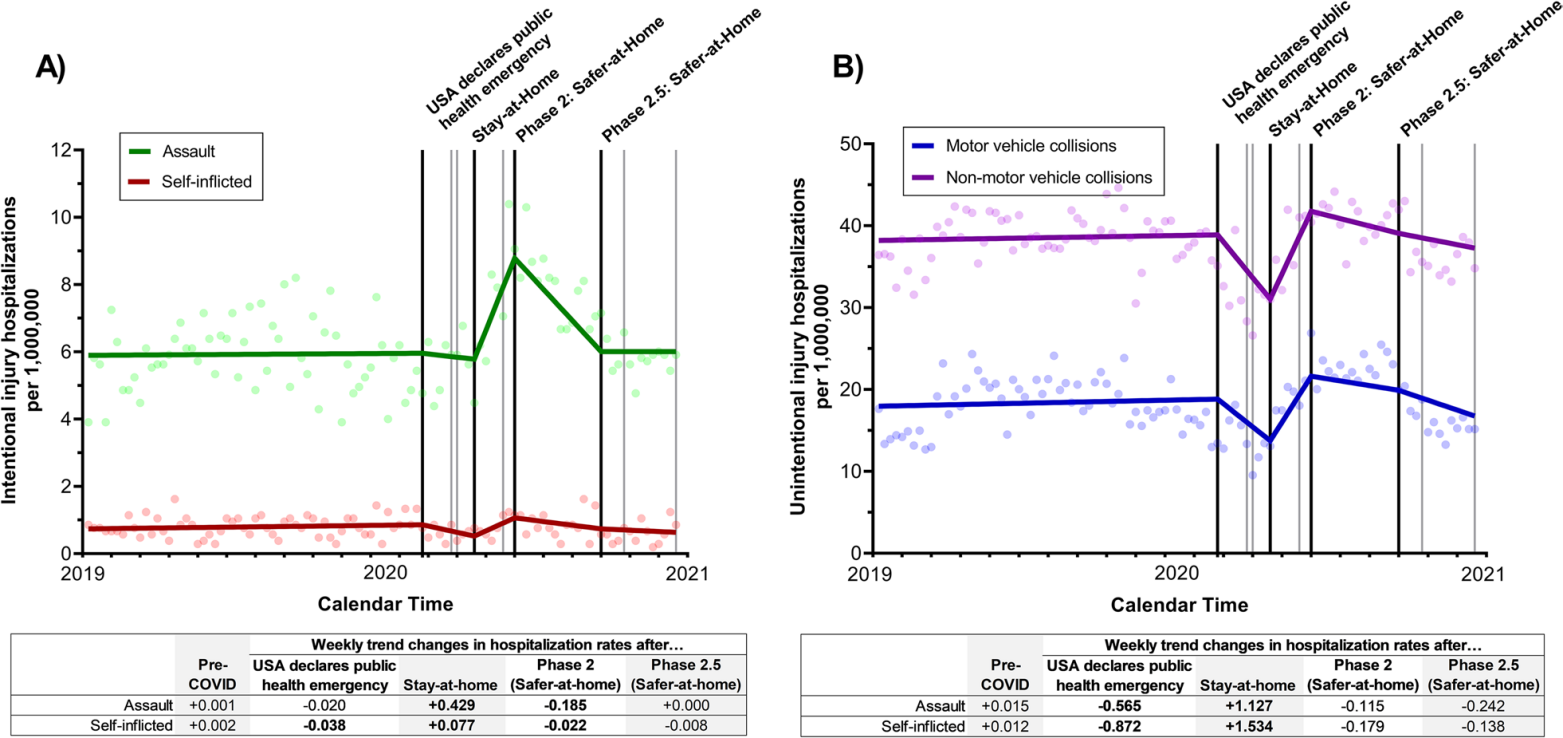
Overall impact of COVID-19 executive orders on the weekly number of trauma admissions for A) intentional and B) unintentional injuries between January 2019 and December 2020 in North Carolina. The black lines represent the timing of the four executive orders assessed in the analyses (the USA declares public health emergency, North Carolina statewide Stay-at-Home order, statewide Phase 2: Safer-at-Home order, and statewide Phase 2.5: Safer-at-Home order); gray lines represent the time of the other COVID-related executive orders. Weekly trend changes in **bold** are statistically significant (p < 0.05)

However, statistically significant changes were seen during several time periods during the first year of the pandemic, Fig. 1. After the statewide Stay-at-Home order was issued, the weekly rate of assault hospitalizations began significantly increasing (weekly trend change = 0.429, 95% CI = 0.349 to 0.509, p < 0.0001) (see Additional file 1: Table S2). Self-inflicted injury hospitalization rates also began increasing after the Stay-at-Home order was issued (weekly trend change = 0.077, 95% CI = 0.040 to 0.115, p = 0.0001). Rates started declining after the Stay-at-Home order was lifted (Phase 2: Safer-at-Home) and by the time Phase 2.5:Safer-at-Home was implemented, both assault (average rate = 6.01, weekly trend change = 0.000, 95% CI = -0.050 to 0.050, p = 0.99) and self-inflicted injury (average rate = 0.68, weekly trend change = -0.008, 95% CI = -0.032 to 0.016, p = 0.51) hospitalization rates had returned to pre-COVID levels, Fig. 1A.

Both unintentional MVCs (weekly trend change = -0.565, 95% CI = -0.968 to -0.163, p = 0.007) and unintentional non-MVC injury hospitalization rates (weekly trend change = -0.872, 95% CI = -1.282 to -0.463, p < 0.001) started to decline after the USA declaration of a public health emergency. Unintentional injury (MVC and non-MVC) hospitalization rates began increasing after the Stay-at-Home orders were implemented (MVC: weekly trend change = 1.127, 95% CI = 0.546 to 1.708, p = 0.0003; non-MVC: weekly trend change = 1.534, 95% CI = 0.910 to 2.157, p < 0.0001), and by the end of 2020 MVC and non-MVC injury hospitalization rates had largely returned to pre-COVID levels (MVC: average rate = 18.08, weekly trend change = -0.242, 95% CI = -0.577 to 0.093, p = 0.16; non-MVC: average rate = 38.03, weekly trend change = -0.138, 95% CI = -0.477 to 0.200, p = 0.43), Fig. 1B.

### Disparities in assault hospitalization rates during COVID-19

Prior to the COVID-19 pandemic, racial/ethnic disparities were seen in North Carolina assault hospitalization rates. Black/African American residents had over 5 times the rate of hospitalizations with assault injuries compared to White, Hispanic/Latino, and other race residents (Black/African American average rate = 16.02; Hispanic/Latino average rate = 3.34; White average rate = 2.79; other race average rate = 2.99). Between when the Stay-at-Home order was issued and when major restrictions were first lifted (Phase 2: Safer at Home), the weekly rate of assault hospitalization rates started increasing dramatically among Black/African American residents (weekly trend change = 1.147, 95% CI = 0.634 to 1.662, p < 0.0001), peaking at an estimated 24.6 assault hospitalizations per 1,000,000 Black/African American residents per week in late May 2020, Fig. 2A. Conversely, relatively small increases in the weekly assault hospitalization rate were seen among Hispanic/Latino (weekly trend change = 0.233, 95% CI = -0.085 to 0.551, p = 0.15), White (weekly trend change = 0.246, 95% CI = 0.137 to 0.356, p < 0.0001), and other race residents (weekly trend change = 0.121, 95% CI = -0.095 to 0.337, p = 0.28) after the Stay-at-Home order was implemented. By the end of 2020, assault hospitalization rates among Black/African Americans leveled off (weekly trend change = 0.094, 95% CI = -0.200 to 0.390, p = 0.53), but appeared to remain elevated compared to pre-pandemic rates (17.53 average weekly rate after Phase 2.5 was implemented vs. 16.02 pre-pandemic).

**Fig. 2:**
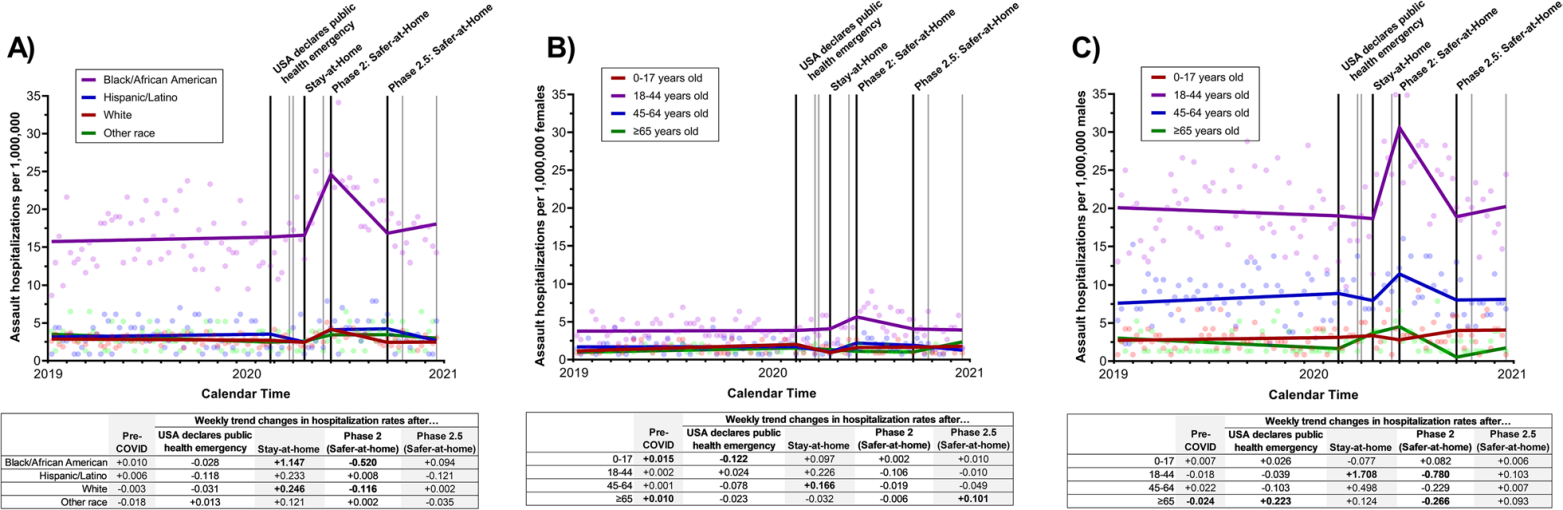
Impact of COVID-19 executive orders on the weekly number of assault admissions between January 2019 and December 2020 in North Carolina, stratified by A) race/ethnicity, B) age group among females, and C) age group among males. The black lines represent the timing of the four executive orders assessed in the analyses (the USA declares public health emergency, North Carolina statewide Stay-at-Home order, statewide Phase 2: Safer-at-Home order, and statewide Phase 2.5: Safer-at-Home order); gray lines represent the time of the other COVID-related executive orders. Weekly trend changes in **bold** are statistically significant (p < 0.05)

Prior to the pandemic, assault hospitalization rates among 18–44-year-old males (average rate = 19.55) were also substantially higher compared to both females (0–17 average rate = 1.59; 18–44 average rate = 3.81; 45–64 average rate = 1.70; ≥ 65 average rate = 1.26) and their other male counterparts (0–17 average rate = 2.92; 45–64 average rate = 8.24; ≥ 65 average rate = 2.34). After Stay-at-Home orders were issued, weekly increases in assault hospitalizations were only observed among 18–44-year-old males, equating to almost 2 additional hospitalizations, per 1,000,000 males 18–44 years old (weekly trend change = 1.708, 95% CI = 0.870 to 2.545, p = 0.0001), Fig. 2C. This increase led to assault hospitalization rates peaking at an estimated 30.34 hospitalizations per 1,000,000 males aged 18–44 in late May 2020. Rates among males ages 18–44, began to decline after the Stay-at-Home order was first lifted (Phase 2: Safer-at-Home, weekly trend change = -0.780, 95% CI = -1.200 to -0.360, p = 0.0004), and returned back to pre-pandemic levels by the end of 2020 (average rate = 19.674; weekly trend change = 0.103, 95% CI = -0.390 to 0.596, p = 0.68).

After the Stay-at-Home order, almost no changes were seen in children (female weekly trend change = 0.097, 95% CI = -0.046 to 0.241, p = 0.19; male weekly trend change = -0.077, 95% CI = -0.287 to 0.131, p = 0.47), 18–44-year-old females (weekly trend change = 0.226, 95% CI = -0.045 to 0.496, p = 0.11), or older adults (female 45–64 weekly trend change = 0.166, 95% CI = 0.015 to 0.316, p = 0.03; female ≥ 65 weekly trend change = -0.032, 95% CI = -0.139 to 0.075, p = 0.56; male 45–64 weekly trend change = 0.498, 95% CI = -0.038 to 1.035, p = 0.07; male ≥ 65 weekly trend change = 0.124, 95% CI = -0.111 to 0.360, p = 0.30), Fig. 2B, C.

### Disparities in unintentional MVC hospitalization rates during COVID-19

Across all racial/ethnic and age/sex groups, the rates of unintentional MVC hospitalizations began declining after the USA declared a public health emergency (Black/African American: weekly trend change = -0.724, 95% CI = -1.272 to -0.176, p = 0.01; Hispanic/Latino: weekly trend change = -0.672, 95% CI = -1.054 to -0.290, p = 0.0009; White: weekly trend change = -0.522, 95% CI = -0.867 to -0.176, p = 0.004; other race: weekly trend change = -0.629, 95% CI = -1.056 to -0.202, p = 0.005), but appeared to return to pre-pandemic levels by the end of 2020, Fig. 3A. Black/African American residents appeared to experience a larger average increase in weekly unintentional MVC hospitalizations after Safer-at-Home was implemented compared to other racial/ethnic groups (Black/African American: weekly trend change = 1.908, 95% CI = 1.091 to 2.725, p < 0.0001; Hispanic/Latino: weekly trend change = 1.178, 95% CI = 0.619 to 1.737, p < 0.0001; White: weekly trend change = 0.858, 95% CI = 0.361 to 1.356, p = 0.001; other race: weekly trend change = 0.886, 95% CI = 0.276 to 1.495, p = 0.005), and by the time Phase 2:Safer-at-Home was implemented, the average weekly MVC hospitalization rate among Black/African American residents was higher than pre-pandemic rates (first week of Safer-at-Home rate = 27.50 vs. pre-pandemic rate = 19.29); rates above pre-pandemic levels were not observed among other racial/ethnic groups.

**Fig. 3:**
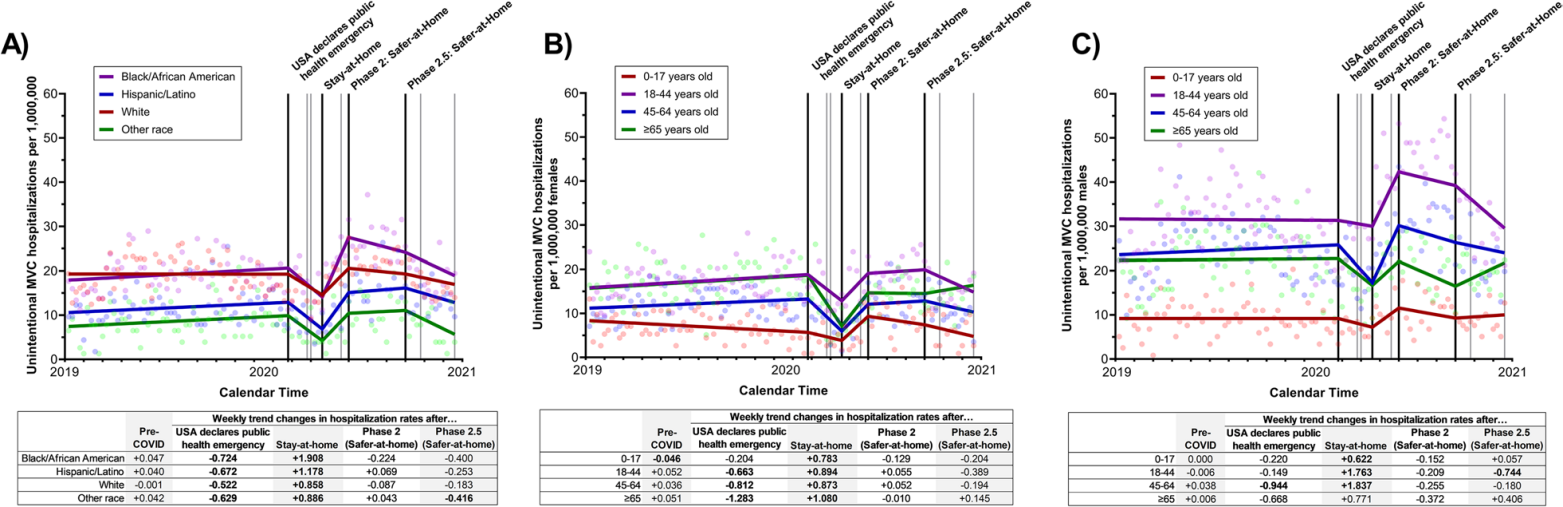
Impact of COVID-19 executive orders on the weekly number of unintentional MVC admissions between January 2019 and December 2020 in North Carolina, stratified by A) race/ethnicity, B) age group among females, and C) age group among males. The black lines represent the timing of the four executive orders assessed in the analyses (the USA declares public health emergency, North Carolina statewide Stay-at-Home order, statewide Phase 2: Safer-at-Home order, and statewide Phase 2.5: Safer-at-Home order); gray lines represent the time of the other COVID-related executive orders. Weekly trend changes in **bold** are statistically significant (p < 0.05)

Among females, compared to pre-pandemic rates, the largest average weekly decline in unintentional MVC hospitalization rates appeared to occur among those ≥ 65 years old (weekly trend change = -1.283, 95% CI = -1.892 to -0.674, p < 0.0001) after the USA declared a public health emergency, although declines were seen among all adult female age groups (18–44 weekly trend change = -0.663, 95% CI = -1.138 to -0.190, p = 0.007; 45–64 weekly trend change:-0.812, 95% CI = -1.165 to -0.459, p < 0.0001); no significant change was seen among those 0–17 years old (weekly trend change = -0.204, 95% CI = -0.151 to 0.558, p = 0.26), Fig. 3B. Overall, unintentional MVC hospitalization rates appeared to return to pre-pandemic levels within a few months among all adult females. Among males, adults 18–44 years old experienced almost no weekly change in unintentional MVC hospitalization rates after the USA declared a public health emergency (weekly trend change = -0.149, 95% CI = -0.987 to 0.690, p = 0.73), yet still saw increasing hospitalization rates after the Stay-at-Home order was issued (weekly trend change = 1.763, 95% CI = 0.513 to 3.014, p = 0.007), Fig. 3C. Unintentional MVC hospitalization rates declined throughout 2020 and reached pre-pandemic rates around the end of 2020 (estimated rate during first week in December 2020 = 31.28 vs. pre-pandemic average rate = 31.49). Among 45–64 year-old males, a sharp decline (weekly trend change = 0.944, 95% CI = -1.564 to -0.324, p = 0.004) then rapid recovery (weekly trend change = 1.837, 95% CI = 0.879 to 2.795, p = 0.0003) to pre-COVID to pre-COVID weekly hospitalization rates by the start of Phase 2: Safer-at-Home was seen. No meaningful changes were seen in unintentional MVC hospitalization rates were seen between 2019 and 2020 between males 0–17 years old or ≥ 65 years old (p > 0.05 for all changes in trends), Fig. 3B.

### Disparities in unintentional non-MVC hospitalization rates during COVID-19

**Fig. 4:**
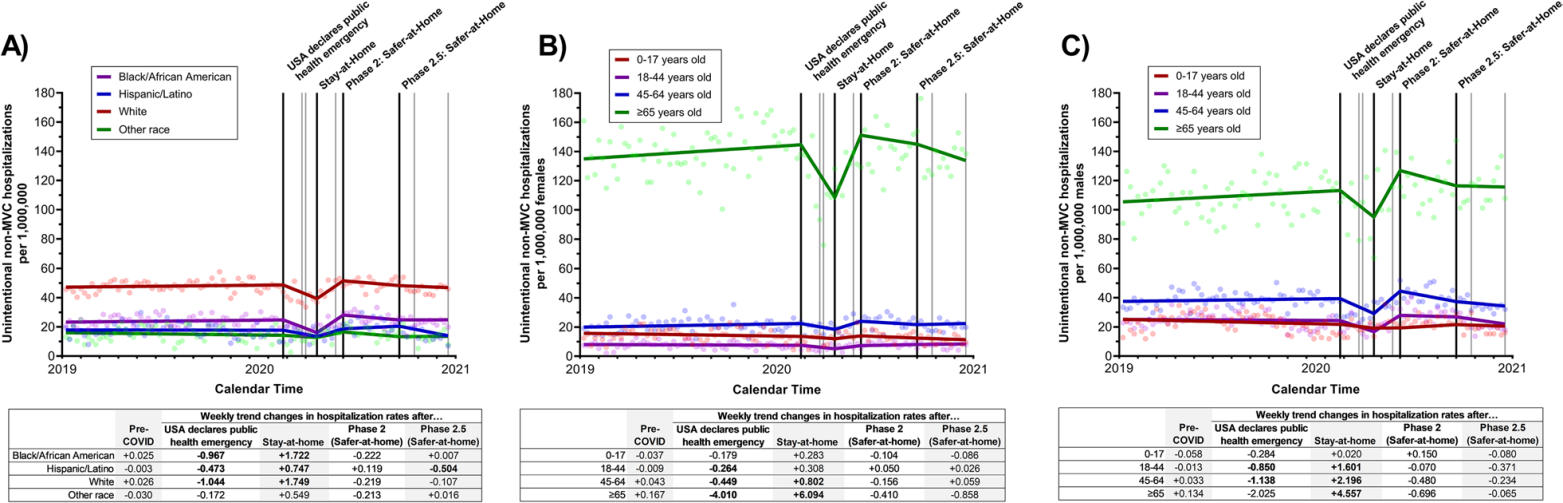
Impact of COVID-19 executive orders on the weekly number of unintentional non-MVC admissions between January 2019 and December 2020 in North Carolina, stratified by A) race/ethnicity, B) age group among females, and C) age group among males. The black lines represent the timing of the four executive orders assessed in the analyses (the USA declares public health emergency, North Carolina statewide Stay-at-Home order, statewide Phase 2: Safer-at-Home order, and statewide Phase 2.5: Safer-at-Home order); gray lines represent the time of the other COVID-related executive orders. Weekly trend changes in **bold** are statistically significant (p < 0.05)

While White residents experienced higher hospitalization rates of non-MVC unintentional injuries prior to the COVID-19 pandemic (White average rate = 47.98; Black/African American average rate = 24.02; Hispanic/Latino average rate = 17.82; other race average rate = 15.11), similar changes in hospitalization rate trends during the COVID-19 pandemic were seen across all racial/ethnic groups, Fig. 4A. Similar to unintentional MVC hospitalizations, after the USA declared a public health emergency, unintentional non-MVC hospitalizations weekly rates declined among Black/African American (weekly trend change = -0.967, 95% CI = -1.442 to -0.492, p = 0.0001), Latino (weekly trend change = -0.473, 95% CI = -0.925 to -0.023, p = 0.04), and White residents (weekly trend change = -1.044, 95% CI = -1.592 to -0.497, p = 0.0003), but rates appeared to return to pre-COVID levels by the time major restrictions were first lifted (Phase 2: Safer-at-Home) among all racial/ethnic groups. No meaningful change was observed among other race residents after the USA declared a public health emergency (weekly trend change = -0.172, 95% CI = -0.827 to 0.483, p = 0.61).

Both older females and males (≥ 65 years old) experienced substantially higher rates of unintentional non-MVC hospitalizations pre-COVID (average rates = 139.79 and 109.33 hospitalizations per 1,000,000, respectively) compared to other age groups (females 0–17 average rate = 14.68; females 18–44 average rate = 7.77; females 45–64 average rate = 21.19; males 0–17 average rate = 23.45; males 18–44 average rate = 24.76; males 45–64 average rate = 38.45). Among adult females 18–64 years-old, only modest declines were seen after the USA declared a public health emergency (18–44 weekly trend change = -0.264, 95% CI = -0.521 to -0.007, p = 0.05; 45–64 weekly trend change = -0.449, 95% CI = -0.704 to -0.194, p = 0.0009) compared to females ≥ 65 years old (weekly trend change = -4.010, 95% CI = -6.166 to -1.855, p = 0.0004); no significant change in weekly trends were seen among females 0–17 (weekly trend change = -0.179, 95% CI = -0.689 to 0.332, p = 0.49), Fig. 4B. A similar pattern was seen among males, with modest declines in weekly rates after the USA declared a public health emergency among males 18–44 (weekly trend change = -0.850, 95% CI = -1.412 to -0.289, p = 0.004) and males 45–64 (weekly trend change = -1.138, 95% CI = -2.117 to -0.159, p = 0.03), with no changes in the weekly rates observed among males 0–17 (weekly trend change = -0.284, 95% CI = -1.019 to 0.451, p = 0.45). Large declines in weekly non-MVC hospitalization rates were also observed among males ≥ 65 years old, although estimates were imprecise (weekly trend change = 2.025; 95% CI = -4.222 to 0.173, p = 0.07), Fig. 4C. Unintentional non-MVC hospitalization rates appeared to return back to pre-COVID weekly rates for all adults (female and male) by the time major restrictions were first lifted (Phase 2: Safer-at-Home).

## Discussion

In a statewide analysis of trauma hospitalizations, we found that the COVID-related policies were associated with changes in assault, self-inflicted, and unintentional injury hospitalization rates. When the statewide Stay-at-Home order was issued in North Carolina, assault hospitalization rates, primarily among Black/African American residents and adult males aged 18–44, increased quickly but then dropped back to pre-pandemic levels once restrictions had been lifted. After the USA declared a public health emergency, both unintentional MVC and non-MVC hospitalization rates decreased across most age groups, with the most substantial changes occurring in older women. Interestingly, men aged 18–44 saw no declines in non-MVC injury hospitalizations after the declaration but still saw the same increase in rates a few months later, with rates not falling to pre-COVID levels until the end of 2020. To the best of our knowledge, this is the first in-depth assessment of changes and disparities in trauma hospitalizations due to a statewide Stay-at-Home order and other COVID-related policies in the USA across race/ethnicity, age, and sex during the pandemic.

Increases in assault hospitalization rates, particularly firearm injuries, during the North Carolina statewide Stay-at-Home orders during the COVID-19 pandemic have been observed in other states in the USA (Hatchimonji et al. 2020; Yeates et al. 2021; Abdallah et al. 2021). In our analysis, we found that assault injury hospitalization rates only increased among Black/African American residents and adults aged 18–44 years old. The disparate effect of Stay-at-Home orders among Black/African American residents, compared to other racial/ethnic groups, may be partially explained by the increased burden of COVID-related financial, mental, and emotional strain (Yancy 2020; Webb Hooper et al. 2020) among a population also at higher risk of experiencing assault. The increased rate of assault hospitalizations among women aged 18–44 indicates that statewide Stay-at-Home orders may have led to an increase in domestic violence, which was both a noted concern (Kaukinen 2020) and has been observed in other studies (Evans et al. 2020; Hsu and Henke 2021). While we did not observe an increase in assaults among children, increases in child abuse have been observed in at least one US-based study (Kovler et al. 2021); it is possible that we were unable to detect a change due to the low baseline rate of assault hospitalizations among this age group. Overall, both our findings and those of other studies suggest that Stay-at-Home orders and other COVID-related policies in the USA, and potentially other countries, had unintended negative consequences and that these were felt more among racial/ethnic minorities, women, and children.

The temporary decreases we saw at the beginning of the pandemic in unintentional injury (MVC and non-MVC) hospitalizations have also been observed in other states in the USA (Chiba et al. 2021; Kamine et al. 2020; Devarakonda et al. 2021; Sherman et al. 2021) and globally (Yasin et al. 2021). Even prior to Stay-at-Home orders in the USA (Fraser et al. 2021), many people began teleworking in the early months of the COVID-19 pandemic (Jacobsen and Jacobsen 2020; Lee et al. 2020), leading to fewer people commuting and fewer MVCs (Devarakonda et al. 2021; Sherman et al. 2021; Sutherland et al. 2020; Fraser Shilling 2020), and likely fewer workplace injuries (unintentional non-MVCs). School closures, which occurred prior to the Stay-at-Home order in North Carolina, may have also led to decreased unintentional hospitalization rates early in the pandemic.

Interestingly, we observed no initial change in the unintentional MVC hospitalization rate among males 18–44 years-old, which is different from the trends we observed in every other sex/age group and inconsistent with previously reported findings of both fewer cars on the road (Jacobsen and Jacobsen 2020; Lee et al. 2020; Fraser Shilling 2020) and fewer MVCs (Chiba et al. 2021; Devarakonda et al. 2021; Sherman et al. 2021; Sutherland et al. 2020) overall during the first several months of the pandemic in the USA (Wellenius et al. 2021) and abroad (Woskie et al. 2021). And while several subgroups in North Carolina saw increases in unintentional MVC hospitalizations after these initial decreases, most stopped once hospitalization rates returned to pre-pandemic levels; among Black/African American residents and men aged 18–44 years old, MVC hospitalization rates rose above pre-COVID levels after the Stay-at-Home order and did not return to pre-COVID levels until the end of 2020. Research is needed to identify potential behavior changes and policy effects that led to these prolonged increases in MVC hospitalizations among these individuals.

We also did not expect to see such substantial declines in unintentional non-MVC injury hospitalizations among older adults—the majority of which were fall-related—in the first few months of the pandemic. While one other US-based study also stratified by age (Chiba et al. 2021), they found no change in unintentional non-MVC hospitalizations among adults ≥ 65 years old. However, hospitals in NCTR have an older patient population, compared to that study (37.1% vs. 16.6% ≥ 65 years old in 2020), and we also assessed weekly rates and allowed trends to change across several COVID-related policies, instead of averaging across the entire COVID-period, which may explain the differences in our findings. This rapid decrease in non-MVC hospitalization rates in North Carolina suggests that older adults, especially older women, may have been avoiding seeking care at a hospital during the pandemic due to fear of COVID-19 infection (Wong et al. 2020). Declines in acute myocardial infarction and stroke hospitalizations (overall and among older adults) during the first few months of the COVID-19 pandemic, illnesses which should not have been impacted by the pandemic or Stay-at-Home orders, have also been reported in the USA (Solomon et al. 2020; Bhambhvani et al. 2021) and Europe (Helal et al. 2021; Nogueira et al. 2021), further suggesting that people have avoided going to the hospital for necessary medical care. Policies and messaging are needed to ensure individuals seek needed urgent care for trauma during outbreaks and pandemics to avoid possible long-term, detrimental effects.

This study has a few limitations. First, the NCTR only includes individuals hospitalized for, died from, or were transferred due to traumatic injuries and therefore only captured a proportion of all traumatic injuries; individuals who were treated in an emergency department (but never admitted) or did not seek care at all would be missed. Given the known disparities in access to care (Dickman et al. 2017) and trauma (Chun Fat et al. 2019; Jager et al. 2019) in the USA, it is likely that we have underestimated the burden of serious traumatic injuries among racial/ethnic minorities. Similarly, only individuals who survived their initial injuries would be sent to a hospital for treatment and could also lead to unequal underestimation; however, we compared trauma changes in rates within racial/ethnic and age/sex groups, which would minimize the effect of underestimation. Third, outside of the COVID-19 pandemic and related policies, there were several, co-occurring nationally recognized events that could also potentially have impacted trauma hospitalization rates (e.g., George Floyd protests which occurred in late May, just after Phase 2: Safer-at-Home began). Thus, our results should be interpreted with some caution. Additionally, we may be limited in our ability to detect meaningful changes because several policies were implemented within a relatively short time, and several time periods had less than two months’ worth of weekly data points. Finally, self-inflicted injury hospitalization rates were too rare to conduct stratified analyses; future studies should utilize databases that capture both emergency department visits and deaths to elucidate concerns regarding self-inflicted injuries and suicide during COVID-19.

## Conclusions

Overall, it appears the Stay-at-Home orders implemented during the COVID-19 pandemic have had unintended consequences that disproportionately impacted racial/ethnic minorities and other marginalized groups in North Carolina, and potentially the USA. Fear of COVID-19 may have also led to decreases in unintentional injury hospitalization rates, particularly among older women, which could have long-term consequences. Given the potential far-reaching adverse impacts of national and statewide policies on racial/ethnic minorities and other high-risk groups, it is crucial for policy makers to anticipate possible negative effects and develop tailored, culturally appropriate approaches to mitigate harms that may disproportionately affect already disadvantaged communities.

## Supplementary Information

**Additional file 1: Table S1**. Dates and descriptions of COVID-19 executive orders in North Carolina. Bolded orders are ones included in analyses. **Table S2**. Segmented Linear Regression Modeling Results, overall. **Table S3**. Segmented Linear Regression Modeling Results, stratified by race/ethnicity. **Table S4**. Segmented Linear Regression Modeling Results, among females and stratified by age. **Table S5**. Segmented Linear Regression Modeling Results, among males and stratified by age.

## Data Availability

Data is available for request from the North Carolina Office of Emergency Medical Services. Researchers will make code available upon request. Please contact Dr. Paula Strassle for access.

## Abbreviations

ED: Emergency department
MVC: Motor vehicle collision
NCTR: North Carolina Trauma Registry
NCOEMS: North Carolina Office of Emergency Medical Services
USA: United States of America
SD: Standard deviation
