# Supplementary material for: Effect of Stay-at-Home orders and other COVID-related policies on trauma hospitalization rates and disparities in the USA: a statewide time-series analysis
Source: Inj Epidemiol. 2022 Nov 21;9:33. doi: 10.1186/s40621-022-00409-2 (PMC9680127; doi:10.1186/s40621-022-00409-2)
Supplement: Supplementary file 1 — Additional file 1: Table S1. Dates and descriptions of COVID-19 executive orders in North Carolina. Bolded orders are ones included in analyses. Table S2. Segmented Linear Regression Modeling Results, overall. Table S3. Segmented Linear Regression Modeling Results, stratified by race/ethnicity. Table S4. Segmented Linear Regression Modeling Results, among females and stratified by age. Table S5. Segmented Linear Regression Modeling Results, among males and stratified by age. [file 40621_2022_409_MOESM1_ESM.docx]

**Supplementary**

**Supplementary Table 1.** Dates and descriptions of COVID-19 executive orders in North Carolina. Bolded orders are ones included in analyses.

| **Order Date** | **Start Date** | **Description** |
| --- | --- | --- |
| **1/31/2020** | **–** | **USA declares public health emergency** |
| 3/10/2020 | – | NC declares state of emergency |
| 3/13/2020 | – | USA declares state of emergency |
| 3/14/2020 | 3/16/2020 | K-12 public schools closed statewide begins   - In effect until 3/30/2020 - Extended to 5/15 on 3/23/20 |
| **3/27/2020** | **3/30/2020**  **(5PM)** | **Statewide Stay-at-Home^a^ order issued**   - Extended to 5/8 on 4/23/20 |
| 5/5/2020 | 5/8/2020  (5PM) | Phase 1 (lessened some Stay-at-Home restrictions)   - In effect until 5/22/20 |
| **5/22/2020** | **5/22/2020**  **(5PM)** | **Phase 2 (Safer-at-Home)^b^ begins; Stay-at-Home order lifted**   - In effect until 6/26/20 - Extended to 7/17 on 6/24/20 - Extended to 8/7 on 7/16/20 - Extended to 9/11 on 8/5/20 |
| **9/1/2020** | **9/4/2020**  **(5PM)** | **Phase 2.5 (Safer-at-Home)^c^**   - In effect until 9/22/2020 |
| 9/30/2020 | 10/2/2020  (5PM) | Phase 3 (Safer-at-Home)^d^   - In effect until 10/23/20 - Extended to 11/13 on 10/21/20 - Extended to 12/4 on 11/10/20 |
| 12/8/2020 | 12/11/2020  (5PM) | Modified Stay-at-Home order^e^   - In effect until 1/8/21 |
| Reference: <https://www.nc.gov/covid-19/covid-19-orders#executive-orders>  ^a^ Stay-at-Home order required people to stay at home except for conducting essential businesses, engaging in outdoor exercise, or assisting family members. The order banned gatherings exceeding 10 people and stipulated people stay at least six feet apart from others  ^b^ Safer-at-Home (Phase 2) order reopened restaurants, childcare businesses, overnight camps, personal care facilities, indoor/outdoor pools, and sporting/entertainment venues that comply with public health requirements (e.g., six feet physical distancing, gatherings limited to 10 people indoors and 25 people outdoors)  ^c^ Safer-at-Home (Phase 2.5) order allowed gatherings to increase to 25 people indoors and 50 people outdoors, museums/aquariums and gyms to open at 50% and 30% capacity respectively, and playgrounds to become available. It also encouraged employers to provide employees with face masks  ^d^ Safer-at-Home (Phase 3) order allowed bars with outdoor seating, outdoor amusement parks, movie theaters, indoor meeting spaces, gaming facilities, and large outdoor facilities to reopen and comply with public health requirements (e.g., social distancing)  ^e^ Modified Stay-at-Home order stated that individuals remain in their place of stay and businesses and facilities close between 10:00pm and 5:00am | | |

**Supplementary Table 2.** Segmented Linear Regression Modeling Results, overall.

|  | **Assault** | | **Self-Inflicted** | | **MVC** | | **Other, Non-MVC** | |
| --- | --- | --- | --- | --- | --- | --- | --- | --- |
|  | **Est.** | **(95% CI)** | **Est.** | **(95% CI)** | **Est.** | **(95% CI)** | **Est.** | **(95% CI)** |
| **Intercept** | 5.896 | (5.697, 6.094) | 0.736 | (0.644, 0.827) | 17.936 | (16.182, 19.689) | 38.192 | (36.746, 39.638) |
| **Pre-COVID Trend Change** | 0.001 | (-0.005, 0.007) | 0.002 | (-0.001, 0.005) | 0.015 | (-0.037, 0.067) | 0.012 | (-0.033, 0.056) |
| **Trend Change after Declaration^a^** | -0.021 | (-0.078, 0.037) | -0.040 | (-0.067, -0.013) | -0.580 | (-1.019, -0.142) | -0.884 | (-1.329, -0.440) |
| **Trend Change after Stay-at-Home** | 0.449 | (0.324, 0.574) | 0.115 | (0.056, 0.174) | 1.692 | (0.800, 2.585) | 2.406 | (1.437, 3.375) |
| **Trend Change after Phase 2: Safer-at-Home** | -0.614 | (-0.716, -0.512) | -0.099 | (-0.148, -0.051) | -1.242 | (-1.987, -0.497) | -1.713 | (-2.511, -0.916) |
| **Trend Change after Phase 2.5: Safer at Home** | 0.185 | (0.110, 0.261) | 0.014 | (-0.022, 0.049) | -0.127 | (-0.663, 0.409) | 0.041 | (-0.510, 0.592) |
| Note: The intercept represents the average rate of hospitalizations, per 1,000,000, at baseline (Jan 2019). To calculate the slope between two time points, add the trend after the time point of interest with all prior weekly trend changes. For example, to estimated slope (week-to-week change) for the rate of assault hospitalizations after the Stay-at-Home order was issued is calculated as (0.001) + (-0.021) + (0.449) = 0.429 and interpreted as an average weekly increase of 0.429 assault hospitalizations, per 1,000,000 residents, after the Stay-at-Home order, but before the Phase 2: Safer-at-Home order.  Abbreviations: Est, estimate; CI, confidence interval  ^a^ Trend after USA declaration of a public health emergency | | | | | | | | |

**Supplementary Table 3.** Segmented Linear Regression Modeling Results, stratified by race/ethnicity.

|  | **Assault** | | **MVC** | | **Other, Non-MVC** | |
| --- | --- | --- | --- | --- | --- | --- |
|  | **Est.** | **(95% CI)** | **Est.** | **(95% CI)** | **Est.** | **(95% CI)** |
|  | **White** | | | | | |
| **Intercept** | 2.868 | (2.585, 3.150) | 19.321 | (17.791, 20.852) | 47.195 | (45.253, 49.136) |
| **Pre-COVID Trend Change** | -0.003 | (-0.011, 0.006) | -0.001 | (-0.046, 0.045) | 0.026 | (-0.036, 0.086) |
| **Trend Change after Declaration^a^** | -0.028 | (-0.112, 0.056) | -0.521 | (-0.897, -0.144) | -1.070 | (-1.664, -0.477) |
| **Trend Change after Stay-at-Home** | 0.277 | (0.106, 0.449) | 1.380 | (0.621, 2.138) | 2.793 | (1.502, 4.084) |
| **Trend Change after Phase 2: Safer-at-Home** | -0.362 | (-0.500, -0.223) | -0.945 | (-1.581, -0.308) | -1.968 | (-3.028, -0.907) |
| **Trend Change after Phase 2.5: Safer at Home** | 0.118 | (0.016, 0.220) | -0.096 | (-0.561, 0.369) | 0.1120 | (-0.620, 0.845) |
|  | **Black/African American** | | | | | |
| **Intercept** | 15.716 | (14.439, 16.993) | 17.881 | (15.789, 19.974) | 23.260 | (21.178, 25.342) |
| **Pre-COVID Trend Change** | 0.010 | (-0.029, 0.049) | 0.047 | (-0.016, 0.111) | 0.025 | (-0.036, 0.087) |
| **Trend Change after Declaration^a^** | 0.018 | (-0.352, 0.389) | -0.771 | (-1.367, -0.175) | -0.992 | (-1.510, -0.474) |
| **Trend Change after Stay-at-Home** | 1.119 | (0.320, 1.919) | 2.632 | (1.361, 3.902) | 2.689 | (1.636, 3.741) |
| **Trend Change after Phase 2: Safer-at-Home** | -1.667 | (-2.321, -1.014) | -2.132 | (-3.168, -1.097) | -1.944 | (-2.822, -1.067) |
| **Trend Change after Phase 2.5: Safer at Home** | 0.614 | (0.155, 1.073) | -0.176 | (-0.890, 0.539) | 0.229 | (-0.404, 0.862) |
|  | **Hispanic/Latino** | | | | | |
| **Intercept** | 3.150 | (2.181, 4.119) | 10.555 | (8.966, 12.145) | 17.929 | (15.948, 19.910) |
| **Pre-COVID Trend Change** | 0.006 | (-0.022, 0.035) | 0.040 | (-0.007, 0.088) | -0.003 | (-0.062, 0.055) |
| **Trend Change after Declaration^a^** | -0.124 | (-0.366, 0.118) | -0.712 | (-1.129, -0.296) | -0.470 | (-0.963, 0.022) |
| **Trend Change after Stay-at-Home** | 0.351 | (-0.139, 0.840) | 1.850 | (0.985, 2.716) | 1.220 | (0.220, 2.220) |
| **Trend Change after Phase 2: Safer-at-Home** | -0.225 | (-0.633, 0.184) | -1.109 | (-1.823, -0.396) | -0.628 | (-1.461, 0.206) |
| **Trend Change after Phase 2.5: Safer at Home** | -0.129 | (-0.425, 0.167) | -0.322 | (-0.839, 0.195) | -0.623 | (-1.227, -0.020) |
|  | **Other race** | | | | | |
| **Intercept** | 3.523 | (2.892, 4.155) | 7.415 | (5.506, 9.323) | 16.019 | (13.434, 18.605) |
| **Pre-COVID Trend Change** | -0.018 | (-0.036, 0.001) | 0.042 | (-0.014, 0.098) | -0.030 | (-0.110, 0.049) |
| **Trend Change after Declaration^a^** | 0.031 | (-0.131, 0.192) | -0.671 | (-1.138, -0.204) | -0.142 | (-0.857, 0.574) |
| **Trend Change after Stay-at-Home** | 0.108 | (-0.229, 0.445) | 1.515 | (0.572, 2.457) | 0.721 | (-0.711, 2.153) |
| **Trend Change after Phase 2: Safer-at-Home** | -0.119 | (-0.392, 0.154) | -0.843 | (-1.625, -0.062) | -0.762 | (-1.996, 0.472) |
| **Trend Change after Phase 2.5: Safer at Home** | -0.037 | (-0.240, 0.166) | -0.459 | (-1.025, 0.107) | 0.229 | (-0.708, 1.167) |
| Note: The intercept represents the average rate of hospitalizations, per 1,000,000, at baseline (Jan 2019). To calculate the slope between two time points, add the trend after the time point of interest with all prior weekly trend changes. For example, to estimated slope for the rate of assault hospitalizations among Black/African American residents after the Stay-at-Home order was issued is calculated as (0.010) + (0.018) + (1.119) = 1.147 and interpreted as an average weekly increase of 1.147 assault hospitalizations, per 1,000,000 Black/African American residents, after the Stay-at-Home order, but before the Phase 2: Safer-at-Home order  Abbreviations: Est, estimate; CI, confidence interval  ^a^ Trend after USA declaration of a public health emergency | | | | | | |

**Supplementary Table 4.** Segmented Linear Regression Modeling Results, among females and stratified by age.

|  | **Assault** | | **MVC** | | **Other, Non-MVC** | |
| --- | --- | --- | --- | --- | --- | --- |
|  | **Est.** | **(95% CI)** | **Est.** | **(95% CI)** | **Est.** | **(95% CI)** |
|  | **Female, 0-17** | | | | | |
| **Intercept** | 1.143 | (0.683, 1.602) | 8.398 | (6.962, 9.835) | 15.783 | (13.748, 17.818) |
| **Pre-COVID Trend Change** | 0.015 | (0.001, 0.029) | -0.046 | (-0.090, -0.002) | -0.037 | (-0.096, 0.022) |
| **Trend Change after Declaration^a^** | -0.137 | (-0.248, -0.026) | -0.158 | (-0.545, 0.230) | -0.142 | (-0.697, 0.414) |
| **Trend Change after Stay-at-Home** | 0.219 | (-0.003, 0.442) | 0.987 | (0.237, 1.738) | 0.462 | (-0.699, 1.624) |
| **Trend Change after Phase 2: Safer-at-Home** | -0.095 | (-0.279, 0.089) | -0.912 | (-1.547, -0.278) | -0.387 | (-1.349, 0.576) |
| **Trend Change after Phase 2.5: Safer at Home** | 0.008 | (-0.119, 0.135) | -0.075 | (-0.575, 0.425) | 0.018 | (-0.728, 0.764) |
|  | **Female, 18-44** | | | | | |
| **Intercept** | 3.760 | (3.021, 4.499) | 15.798 | (13.883, 17.714) | 8.043 | (6.916, 9.170) |
| **Pre-COVID Trend Change** | 0.002 | (-0.022, 0.025) | 0.052 | (-0.006, 0.109) | -0.009 | (-0.042, 0.024) |
| **Trend Change after Declaration^a^** | 0.022 | (-0.195, 0.240) | -0.715 | (-1.232, -0.199) | -0.255 | (-0.535, 0.025) |
| **Trend Change after Stay-at-Home** | 0.202 | (-0.216, 0.619) | 1.557 | (0.472, 2.643) | 0.572 | (0.003, 1.142) |
| **Trend Change after Phase 2: Safer-at-Home** | -0.332 | (-0.684, 0.019) | -0.839 | (-1.732, 0.053) | -0.258 | (-0.733, 0.217) |
| **Trend Change after Phase 2.5: Safer at Home** | 0.096 | (-0.182, 0.375) | -0.444 | (-1.080, 0.192) | -0.024 | (-0.367, 0.319) |
|  | **Female, 45-64** | | | | | |
| **Intercept** | 1.676 | (1.288, 2.063) | 11.157 | (9.811, 12.504) | 19.909 | (18.837, 20.980) |
| **Pre-COVID Trend Change** | 0.001 | (-0.011, 0.013) | 0.036 | (-0.005, 0.077) | 0.043 | (0.011, 0.075) |
| **Trend Change after Declaration^a^** | -0.079 | (-0.197, 0.039) | -0.848 | (-1.232, -0.464) | -0.492 | (-0.770, -0.213) |
| **Trend Change after Stay-at-Home** | 0.244 | (-0.001, 0.488) | 1.685 | (0.865, 2.504) | 1.251 | (0.675, 1.828) |
| **Trend Change after Phase 2: Safer-at-Home** | -0.185 | (-0.368, -0.002) | -0.821 | (-1.495, -0.148) | -0.958 | (-1.434, -0.483) |
| **Trend Change after Phase 2.5: Safer at Home** | -0.030 | (-0.154, 0.094) | -0.246 | (-0.718, 0.225) | 0.215 | (-0.140, 0.569) |
|  | **Female, 65+** | | | | | |
| **Intercept** | 0.964 | (0.663, 1.265) | 15.653 | (13.195, 18.112) | 134.787 | (125.370, 144.204) |
| **Pre-COVID Trend Change** | 0.010 | (0.001, 0.018) | 0.051 | (-0.024, 0.127) | 0.167 | (-0.111, 0.444) |
| **Trend Change after Declaration^a^** | -0.033 | (-0.120, 0.054) | -1.334 | (-2.002, -0.667) | -4.177 | (-6.526, -1.828) |
| **Trend Change after Stay-at-Home** | -0.009 | (-0.188, 0.170) | 2.363 | (1.125, 3.600) | 10.104 | (5.323, 14.884) |
| **Trend Change after Phase 2: Safer-at-Home** | 0.026 | (-0.097, 0.150) | -1.090 | (-2.200, 0.020) | -6.504 | (-10.487, -2.522) |
| **Trend Change after Phase 2.5: Safer at Home** | 0.107 | (0.024, 0.191) | 0.155 | (-0.732, 1.042) | -0.448 | (-3.310, 2.415) |
| Note: The intercept represents the average rate of hospitalizations, per 1,000,000, at baseline (Jan 2019). To calculate the slope between two time points, add the trend after the time point of interest with all prior weekly trend changes. For example, to estimated slope for the rate of assault hospitalizations among females 18-44 years old after the Stay-at-Home order was issued is calculated as (0.002) + (0.022) + (0.202) = 0.226 and interpreted as an average weekly increase of 0.226 assault hospitalizations, per 1,000,000 females 18-44 years old, after the Stay-at-Home order, but before the Phase 2: Safer-at-Home order  Abbreviations: Est, estimate; CI, confidence interval  ^a^ Trend after USA declaration of a public health emergency | | | | | | |

**Supplementary Table 5.** Segmented Linear Regression Modeling Results, among males and stratified by age.

|  | **Assault** | | **MVC** | | **Other, Non-MVC** | |
| --- | --- | --- | --- | --- | --- | --- |
|  | **Est.** | **(95% CI)** | **Est.** | **(95% CI)** | **Est.** | **(95% CI)** |
|  | **Male, 0-17** | | | | | |
| **Intercept** | 2.723 | (2.234, 3.213) | 9.194 | (7.299, 11.089) | 25.199 | (22.751, 27.647) |
| **Pre-COVID Trend Change** | 0.007 | (-0.008, 0.022) | 0.000 | (-0.057, 0.057) | -0.058 | (-0.136, 0.019) |
| **Trend Change after Declaration^a^** | 0.019 | (-0.131, 0.168) | -0.220 | (-0.686, 0.246) | -0.226 | (-1.023, 0.571) |
| **Trend Change after Stay-at-Home** | -0.103 | (-0.430, 0.224) | 0.842 | (-0.076, 1.761) | 0.304 | (-1.453, 2.061) |
| **Trend Change after Phase 2: Safer-at-Home** | 0.159 | (-0.106, 0.424) | -0.774 | (-1.553, 0.004) | 0.130 | (-1.336, 1.596) |
| **Trend Change after Phase 2.5: Safer at Home** | -0.076 | (-0.266, 0.113) | 0.209 | (-0.352, 0.791) | -0.230 | (-1.259, 0.799) |
|  | **Male, 18-44** | | | | | |
| **Intercept** | 20.100 | (17.882, 22.318) | 31.664 | (28.517, 34.810) | 25.152 | (22.693, 27.611) |
| **Pre-COVID Trend Change** | -0.018 | (-0.087, 0.051) | -0.006 | (-0.102, 0.090) | -0.013 | (-0.086, 0.059) |
| **Trend Change after Declaration^a^** | -0.021 | (-0.663, 0.620) | -0.143 | (-1.054, 0.796) | -0.837 | (-1.449, -0.225) |
| **Trend Change after Stay-at-Home** | 1.747 | (0.446, 3.048) | 1.912 | (-0.030, 3.854) | 2.451 | (1.206, 3.696) |
| **Trend Change after Phase 2: Safer-at-Home** | -2.488 | (-3.557, -1.418) | -1.972 | (-3.570, -0.374) | -1.671 | (-2.710, -0.633) |
| **Trend Change after Phase 2.5: Safer at Home** | 0.883 | (0.100, 1.665) | -0.535 | (-1.650, 0.579) | -0.301 | (-1.049, 0.447) |
|  | **Male, 45-64** | | | | | |
| **Intercept** | 7.577 | (6.287, 8.866) | 23.532 | (20.825, 26.238) | 37.467 | (34.417, 40.517) |
| **Pre-COVID Trend Change** | 0.022 | (-0.017, 0.061) | 0.038 | (-0.044, 0.121) | 0.033 | (-0.064, 0.129) |
| **Trend Change after Declaration^a^** | -0.125 | (-0.508, 0.258) | -0.982 | (-1.654, -0.311) | -1.171 | (-2.228, -0.113) |
| **Trend Change after Stay-at-Home** | 0.601 | (-0.232, 1.435) | 2.781 | (1.338, 4.225) | 3.334 | (0.903, 5.766) |
| **Trend Change after Phase 2: Safer-at-Home** | -0.727 | (-1.408, -0.045) | -2.092 | (-3.333, -0.851) | -2.676 | (-4.695, -0.657) |
| **Trend Change after Phase 2.5: Safer at Home** | 0.236 | (-0.232, 0.704) | 0.075 | (-0.798, 0.948) | 0.246 | (-1.102, 1.593) |
|  | **Male, 65+** | | | | | |
| **Intercept** | 3.039 | (2.261, 3.817) | 22.314 | (19.693, 24.934) | 105.298 | (97.276, 113.319) |
| **Pre-COVID Trend Change** | -0.024 | (-0.046, -0.001) | 0.006 | (-0.075, 0.088) | 0.134 | (-0.108, 0.376) |
| **Trend Change after Declaration^a^** | 0.247 | (0.048, 0.446) | -0.674 | (-1.438, 0.090) | -2.159 | (-4.546, 0.228) |
| **Trend Change after Stay-at-Home** | -0.099 | (-0.476, 0.278) | 1.439 | (-0.208, 3.085) | 6.582 | (1.526, 11.638) |
| **Trend Change after Phase 2: Safer-at-Home** | -0.390 | (-0.691, -0.090) | -1.143 | (-2.489, 0.203) | -5.253 | (-9.325, -1.181) |
| **Trend Change after Phase 2.5: Safer at Home** | 0.359 | (0.106, 0.612) | 0.778 | (-0.155, 1.710) | 0.631 | (-2.326, 3.588) |
| Note: The intercept represents the average rate of hospitalizations, per 1,000,000, at baseline (Jan 2019). To calculate the slope between two time points, add the trend after the time point of interest with all prior weekly trend changes. For example, to estimated slope for the rate of assault hospitalizations among males 18-44 years old after the Stay-at-Home order was issued is calculated as (-0.018) + (-0.021) + (1.747) = 1.708 and interpreted as an average weekly increase of 1.708 assault hospitalizations, per 1,000,000 males 18-44 years old, after the Stay-at-Home order, but before the Phase 2: Safer-at-Home order  Abbreviations: Est, estimate; CI, confidence interval  ^a^ Trend after USA declaration of a public health emergency | | | | | | |
